# Respiratory effects of rectus sheath block in patients undergoing major upper abdominal surgery: a randomized controlled trial

**DOI:** 10.1186/s12871-026-03666-6

**Published:** 2026-02-26

**Authors:** Noha Yahia Mohammd El-hagagy, Beshoy Baligh Bolis Gayeed, Mohamed Mohamed Abdel-Latif

**Affiliations:** 1 Lecturer of Anesthesia and Intensive Care Department, Faculty of Medicine, Assiut University, Assiut, Egypt; 2 Resident at Department of Anesthesia and Intensive Care, Faculty of Medicine, Assiut University, Assiut, Egypt; 3 Professor of Anesthesia and Intensive Care Department, Faculty of Medicine, Assiut University, Assiut, Egypt

**Keywords:** Diaphragmatic inspiratory amplitude, Rectus sheath block, Upper abdominal surgery, Postoperative pulmonary complications

## Abstract

**Background:**

Postoperative pulmonary complications remain a major concern after upper abdominal surgery and are often exacerbated by either inadequate pain control or opioid-based analgesia. We evaluated the analgesic efficacy and respiratory effects of ultrasound-guided RSB in patients undergoing upper abdominal surgery.

**Methods:**

In this prospective, randomized, controlled trial, 60 patients aged 18–62 years, with ASA I or II status, who underwent upper abdominal surgery under general anesthesia, were enrolled and divided into two groups (30 patients each). RSB Group received ultrasound-guided rectus sheath block after induction of general anesthesia with 40 ml volume of 0.25% bupivacaine and 8 mg dexamethasone (half the volume on each side). The control group received standard anesthesia care without RSB. Pain scores, opioid consumption, diaphragmatic inspiratory amplitude (DIA), and pulmonary function tests were assessed in the immediate postoperative period.

**Results:**

Diaphragmatic inspiratory amplitude decreased significantly from baseline at 6, 12, and 24 h postoperatively in both groups (P value < 0.001) with no significant intergroup differences. Forced vital capacity (FVC), forced expiratory volume in first second (FEV1) and Peak expiratory flow rate (PEFR) declined postoperatively in both groups but remained comparable between groups. The RSB group demonstrated significantly lower early postoperative pain scores (*P* = 0.007), delayed time to first rescue analgesia, reduced total opioid consumption (*P* < 0.001), and higher patient satisfaction (*P* = 0.041) with reduced incidence of side effects compared with controls.

**Conclusion:**

RSB provided effective opioid-sparing analgesia without impairing pulmonary function. Its simplicity, safety, and compatibility with enhanced recovery after surgery (ERAS) protocols highlight its role as a valuable adjunct to multimodal analgesia in upper abdominal surgery.

## Introduction

Postoperative pulmonary complications continue to be an important risk after major abdominal surgery and account for approximately 25% of postoperative deaths occurring within 6 days of surgery. After open upper abdominal surgery, patients routinely develop a restrictive respiratory deficit characterized by a severe reduction (50–60%) in vital capacity and a lesser reduction (20%) in functional residual capacity, which does not fully recover within the first postoperative week, regardless of anesthetic technique. Multiple factors contribute to postoperative pulmonary impairment following abdominal surgery, such as incision size and location, the level of pain experienced, and the extent of diaphragmatic dysfunction [1, 2]. 

Ultrasound is now a well-established method for qualitatively assessing diaphragmatic motion under both normal and pathological conditions. In particular, M-mode sonography serves as a reliable tool for identifying anatomical and functional abnormalities of the diaphragm [2, 3]. When combined with spirometry, it provides a useful adjunct to functional respiratory assessment [4, 5]. Several studies have evaluated postoperative pain-related respiratory compromise and effects of different analgesic modalities on lung functions [6]. 

A major proportion of pain experienced by patients undergoing abdominal surgeries is due to somatic pain signals derived from the abdominal wall. The central portion of anterior abdominal wall components (skin, muscles, and parietal peritoneum) is innervated by sensory neurons branching from the anterior rami of spinal nerves T7 to T11. These neurons lie between the rectus abdominis muscle and the posterior rectus sheath and pierce the rectus muscle close to the midline. The tendinous intersections of the rectus muscle do not fuse with the posterior rectus sheath, thereby allowing the injectate to spread cephalo-caudally within this potential space [7]. Rectus sheath block(RSB) has been described for midline abdominal incisions (gynecological surgery) [8]. As visceral pain becomes attenuated by the 2nd postoperative day, rectus sheath block can also be administered for midline laparotomy [9]. 

We hypothesized that effective pain control using ultrasound-guided Rectus Sheath Block could preserve diaphragmatic and respiratory function in patients undergoing upper major abdominal surgeries. The primary objective was to evaluate diaphragmatic excursion during the first postoperative 24 h after upper major abdominal surgeries by M-mode ultrasonography. The secondary objectives were to evaluate the pulmonary function tests, the verbal rating scale (VRS), the first rescue analgesia time, the total analgesic requirement in the first postoperative 24 h, patients’ satisfaction at the end of the study, and side effects in the studied groups.

### Patients and methods

#### Enrollment and Eligibility

This prospective, single-center, randomized controlled double-blinded trial was conducted at Assiut University Hospital. The protocol was approved by the Faculty of Medicine Ethics Committee, Assiut University (IRB:17100594), registered on ClinicalTrials.gov (ID: NCT03725527) on 09/29/2018, and conducted in accordance with the Consolidated Standards of Reporting Trials (CONSORT) guidelines and the Declaration of Helsinki. Sixty patients of both sexes were studied between March 2024 and November 2024.aged between 18 and 62 years, classified as American Society of Anesthesiologists (ASA) grade I-II, body mass index (BMI)< 30 kg/m^2^, and undergoing elective upper abdominal surgery under general anesthesia were enrolled after providing written informed consent. Exclusion criteria included infection at the block site, coagulopathy, known allergy to local anesthetics, pregnancy, impaired pulmonary reserve, recent respiratory tract infection (within the last 2 weeks), significant cardiac disease, renal impairment, pre-existing neurological deficits, or psychiatric disorders.

#### Randomization and blindness

Internet websites (http://www.random.org/) were used for random allocation, and each patients’ code was kept in an opaque sealed envelope. Patients were randomly allocated with 1:1 allocation ratio into two groups in a parallel manner: RSB Group (*n* = 30): Patients received ultrasound-guided rectus sheath block (RSB) performed after induction of general anesthesia and before surgery, and Control Group (*n* = 30): Patients received general anesthesia with standard care without the block. The anesthesiologist performing the block was not blinded to group allocation; however, patients, and outcome assessors were blinded to group assignment.

#### Study protocol

During the preoperative visit, the patients’ Medical and surgical histories were taken, a clinical examination was performed, and routine laboratory investigations were conducted. Each patient was informed about the study protocol, procedures, and any potential side effects; demographic data were collected and recorded. Each patient was instructed in how to describe their pain using the verbal rating pain scale (VRS) “(Ranging from 0 = no pain, 1 = mild pain, 2 = moderate pain, 3 = severe pain, and lastly 4 = excruciating pain)” [10], and in how to perform Spirometric measurements.

All patients fasted overnight and received oral premedication with alprazolam 0.25 mg and ranitidine 150 mg the evening before and 2 h prior to surgery. Spirometric measurements were undertaken approximately 1 h before anesthesia induction. All tests were performed at the bedside with the patients in a sitting or semi-recumbent position. At least 3 acceptable measurements were done to meet the European Respiratory Society criteria for reproducibility [11] Forced vital capacity (FVC), forced expiratory volume in first second (FEV1), and peak expiratory flow rate (PEFR) were measured using hand-held spirometer (One-flow^®^, Clement Clarke, U.K). At each assessment, the largest values of FEV1, FVC, and PEFR were recorded. spirometry was repeated at 6,12, and 24 h postoperatively.

### M-mode sonography for evaluation of diaphragmatic movement

Sonographic assessment was performed in the semi-recumbent position with the head of the bed elevated to 45°, immediately following spirometric testing. The examination was conducted by an experienced physician blinded to pulmonary function test (PFT) results, under radiologist supervision, using a real-time sector-scanning sonographic system equipped with a 3.5-MHz phased-array probe (Vivid-I, GE Medical Systems, Shanghai, China). Patients were instructed to breathe quietly, then perform maximal inhalation followed by a forceful and rapid exhalation until no further air could be expelled. These maneuvers were practiced several times prior to the assessment. The probe was positioned along the anterior axillary line in the intercostal space and angled medially, cephalic, and dorsally to allow the ultrasound beam to visualize the posterior dome of the right diaphragm at a nearly perpendicular angle [1]. The probe position was gradually adjusted caudally across successive intercostal spaces until the descending lung shadow disappeared from the ultrasound monitor. All patients were examined along the longitudinal axis of the intercostal space, using the liver as an acoustic window. The diaphragm was visualized first in the B-mode as an echogenic line located between the lung and liver, and its motion during respiratory maneuvers was subsequently evaluated using M-mode sonography. The M-mode tracing was displayed on the screen at a sweep speed of 1.25 cm/s and digitally stored on the system’s hard drive. Diaphragmatic motion was assessed at the posterior surface of the diaphragm, with diaphragmatic inspiratory amplitude (DIA, in centimeters) measured as the distance between echogenic lines on frozen images, using calipers positioned at the center of the lines. Three consecutive sonographic assessments were obtained, and the highest value of the three measurements was recorded.

#### Intraoperative

Routine monitoring included electrocardiogram (ECG), pulse oximetry, non-invasive blood pressure, end-tidal carbon dioxide, and temperature. The data were recorded before induction, after induction, and then every 30 min until the end of surgery.

Anesthetic protocol was standardized for all patients and consisted of an intravenous induction with Fentanyl 2 mcg/kg, propofol 2–3 mg/kg, and lidocaine 2 mg/kg. Cis-atracurium 0.15 mg/kg was given to facilitate endotracheal intubation. Anesthesia and muscle relaxation were maintained with Isoflurane in 50% oxygen/air mixture, and regular use of cis-atracurium 0.03 mg/kg and mechanical ventilation by volume-controlled mode to maintain normocapnia. Hemodynamics were adjusted accordingly with a goal of 80–120% baseline noninvasive mean arterial pressure. Fentanyl 1 mcg/kg IV was administered for any intraoperative increase in the heart rate (HR) or mean arterial pressure (MAP) above 20% of baseline. All patients received 60 mg ketorolac intraoperative and before skin closure, 1 gm paracetamol is given intravenously. Following completion of the surgical procedure, muscle relaxation was reversed with Neostigmine 50 µg/kg and Atropine 20 µg/kg. The patients were extubated awake and transported to the post-anesthesia care unit (PACU).

### Ultrasound-guided rectus sheath block (RSB)

After induction of general anesthesia and before surgery, RS blocks were performed in patients in the RSB Group by an experienced investigator under dynamic ultrasound guidance (M-Turbo, Sonosite Inc., Bothell, WA, USA). Broad and linear array ultrasound probe was placed in the axial plane. The technique was performed with complete aseptic precautions while the patient was in a supine position. The high-frequency (11 MHz) linear array ultrasound probe was positioned at the level of the umbilicus immediately lateral in a transverse plane to the abdominal wall, enabling rectus muscle identification and localization of the hyperechoic matching lines deep to it (posterior rectus sheath and fascia transversalis). A 16-G, 8-cm Tuohy needle (Portex; Smiths Medical International Ltd, Kent, UK) was directed at approximately 45 degrees to the skin. The needle was then positioned in plane and directly under the ultrasound probe in a medial to lateral orientation and then advanced through the subcutaneous tissue to pierce through the anterior rectus sheath. The needle was then further advanced through the body of the rectus muscle until it reaches the plane between the rectus muscle and the posterior rectus sheath. Upon reaching this potential space, after careful aspiration, installation of (19 ml) of 0.25% bupivacaine and 4 mg dexamethasone(1 ml) (total volume of 20 ml) was injected to dissect the posterior rectus sheath on each side. The procedure was repeated on the other side under ultrasound guidance.

Postoperative: A standardized analgesic regimen was prescribed in the postoperative period. All patients received paracetamol 1 gram every 6 h as routine analgesia. Patients received rescue nalbuphine analgesia if VRS scores were ≥ 2 despite the block (breakthrough pain).

The adverse effects in PACU also were assessed: hypotension (decrease in basal mean arterial blood pressure by 20%) was treated with IV fluid, bradycardia (defined by decrease in basal heart rate by 20%) was treated by IV atropine 0.02 mg/kg, respiratory depression (the SpO2 < 95% and need O2 supplementation), and postoperative nausea and vomiting (PONV) was treated by ondansetron 0.1 mg/kg IV, and any complications related to the block were recorded.

#### Assessment Parameters

Patient demographics and clinical data, including age, weight, gender, ASA class, BMI, and duration of surgery, were recorded.

##### Diaphragmatic M- mode ultrasonography

 DIA was recorded preoperatively and then at 6, 12, and 24 h postoperatively (primary outcome).

##### Respiratory function

 FVC, FEV1, and PEFR were recorded preoperatively and at 6, 12, and 24 h postoperatively.

##### Postoperative pain assessment

 VRS scores were recorded. on admission to PACU (baseline), and 2, 4, 6, 12, and 24 h postoperatively. Patients received rescue analgesia if requested and if VRS scores were ≥ 2.

##### Intraoperative fentanyl consumption, time to first request of rescue analgesia, and the total consumption of postoperative nalbuphine 

Administered for breakthrough pain were recorded.

##### Assessment of patient satisfaction

 Postoperatively, the level of patient satisfaction was measured using a five-point Likert scale (1 = extremely unsatisfied, 2 = unsatisfied, 3 = neutral, 4 = satisfied, and 5 = extremely satisfied).

##### Vital signs

 Noninvasive blood pressure, heart rate, and oxygen saturation by pulse oximeter were recorded before the block (baseline) and every 30 min till the end of the operation. And postoperative at PACU and at 30 min, 2, 4, 6, 12, and 24 h postoperatively.

##### Adverse effects

 Any perioperative adverse event was treated and recorded. These could include hypotension, bradycardia, nausea, vomiting, Allergic reaction, respiratory depression, or complications related to the technique of the block (such as needle trauma or inadvertent intravascular injury).

#### Sample size calculation

The sample size calculation was performed using G*Power 3.1.9.7 (Universitat Kiel, Germany) based on DIA in the first postoperative day (at 24 h) as a primary outcome. Based on previous study [1] they reported DIA in the first postoperative day to be 0.99 ± 0.3, mean ± SD in order to detect an improvement of 0.4 in the DIA, we need to include 26 patients per group with α error = 0.05, power of 0.95 and 1:1 allocation, two tailed study. Another four patients were added to each group to overcome dropout. Therefore, 30 patients were recruited in each group.

#### Statistical analysis

Statistical analysis was done by SPSS v26 (IBM Inc., Chicago, IL, USA). The Shapiro-Wilks test and histograms were used to evaluate the normality of the distribution of data. Quantitative parametric variables were presented as mean and standard deviation (SD) and compared between the two groups utilizing unpaired Student’s t-test, and Paired samples t-test to compare with baseline. Quantitative non-parametric data were presented as median and interquartile range (IQR) and were analyzed by Mann Whitney-test, and Willcoxon Signed Rank test to compare with baseline. Qualitative variables were presented as frequency and percentage (%) and were analyzed utilizing the Chi-square test or Fisher’s exact test when appropriate. A two tailed P value < 0.05 was considered statistically significant.

## Results

**Fig. 1:**
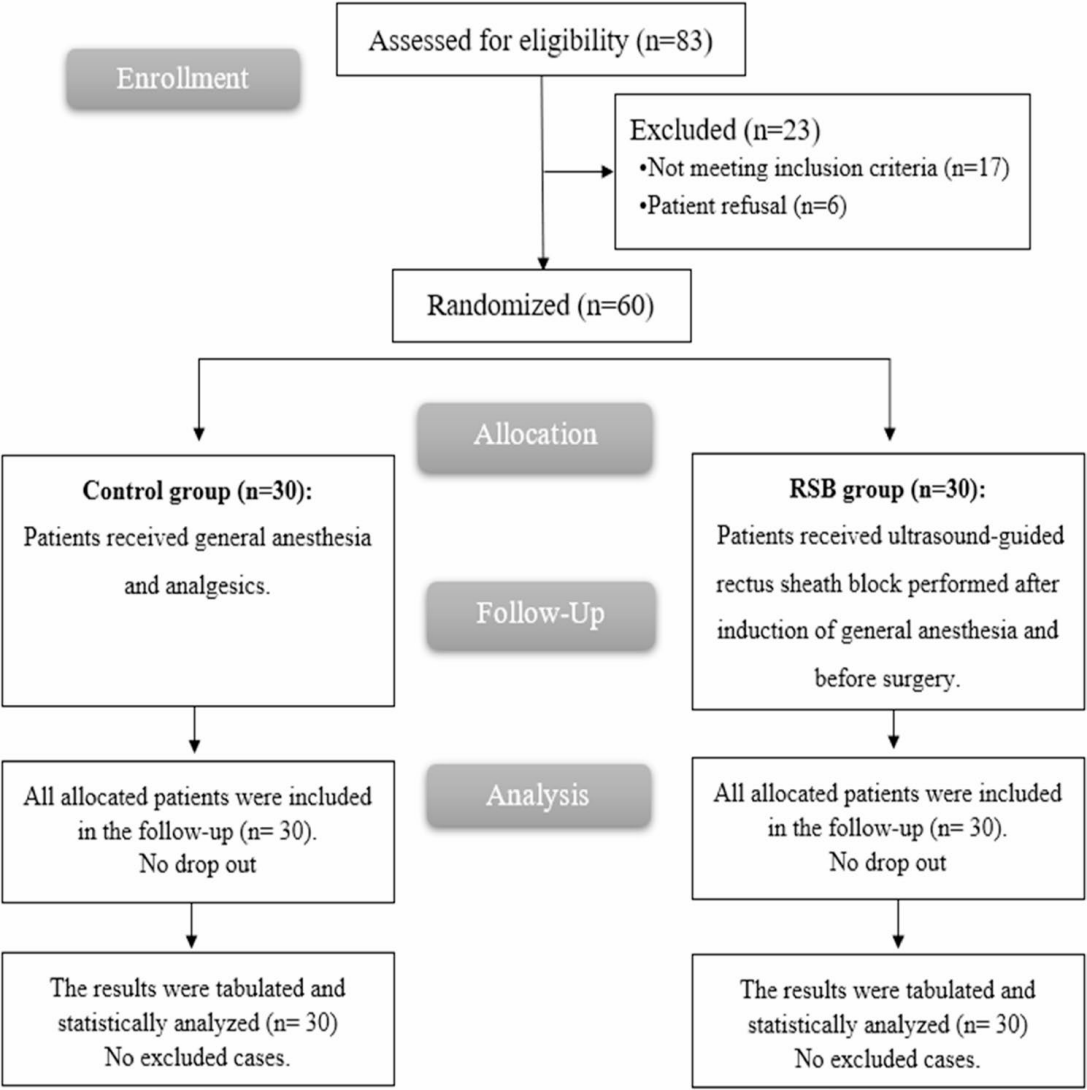
CONSORT flowchart of the enrolled patients

A total of 83 patients were assessed for eligibility, of whom 17 patients did not meet inclusion criteria, and 6 declined participation. The remaining 60 patients were randomized and analyzed (Fig. 1). Baseline demographic and clinical characteristics were similar between the RSB and control groups (Table 1).

**Table 1:** Demographic and clinical characteristics of the study groups

		Control group (*n* = 30)	RSB group (*n* = 30)	*P* value
	Mean ± SD	40.77 ± 11.92	43.5 ± 13.64	0.412
Age (years)				
Sex	**Male**	22 (73.33%)	20 (66.67%)	0.573
	**Female**	8 (26.67%)	10 (33.33%)	
Weight (kg)	**Mean ± SD**	71.37 ± 8.67	70.67 ± 7.25	0.736
Height (cm)	**Mean ± SD**	167.5 ± 7.04	167.87 ± 5.76	0.826
BMI (kg/m2)	**Mean ± SD**	25.47 ± 2.9	25.11 ± 2.51	0.603
ASA physical status	**I**	20 (66.67%)	17 (56.67%)	0.426
	**II**	10 (33.33%)	13 (43.33%)	
Duration of surgery (min)	**Mean ± SD**	193.33 ± 12.82	187.5 ± 13.57	0.092
Need of intraoperative fentanyl		4 (13.33%)	2 (6.67%)	0.671
Intraoperative fentanyl consumption (µg)	**Mean ± SD**	72.5 ± 12.58	65 ± 7.07	0.492
	**Range**	60–90	60–70	
First request time of rescue analgesia (h)	**Mean ± SD**	3.03 ± 0.89	5.1 ± 0.92	< 0.001*
	**Range**	2–4	4–6	
Total nalbuphine consumption (mg)	**Mean ± SD**	9.2 ± 2.61	6.8 ± 1.86	< 0.001*
	**Range**	4–12	4–8	
PONV(n)		9 (30%)	2 (6.67%)	0.042*
Respiratory depression(n)		0 (0%)	0 (0%)	---
Needle trauma(n)		0 (0%)	0 (0%)	---
Allergic reaction(n)		0 (0%)	0 (0%)	---
Extremely satisfied(n)		4 (13.33%)	12 (40%)	0.041*
Satisfied(n)		10 (33.33%)	11 (36.67%)	0.041*
Neutral(n)		11 (36.67%)	7 (23.33%)	0.041*
Unsatisfied(n)		3 (10%)	0 (0%)	0.041*
Extremely unsatisfied(n)		2 (6.67%)	0 (0%)	0.041*

Data are presented as mean ± SD or as numbers. *Abbreviations*: *SD *standard deviation, American Society of Anesthesiologists (*ASA*), body mass index (*BMI*), number (n). *P* < 0.05 indicates statistically significant differences

Compared with preoperative baseline values, the mean DIA decreased significantly at 6, 12, and 24 h postoperatively in both groups (*P* < 0.001), with the greatest reduction observed at 24 h. No significant differences in DIA were detected between the groups at any time point. (Table 2)

**Table 2:** Ultrasound diaphragmatic excursion and pulmonary function of the studied groups

	Control group (*n* = 30)	RSB group (*n* = 30)	*P*-value1	*P*-value2	*P*-value3
DIA (mm)					
Baseline	23.00 ± 1.17	22.67 ± 1.35	0.311		
6 h	19.00 ± 2.26	19.30 ± 2.02	0.590	0.000*	0.000*
12 h	18.60 ± 2.06	18.33 ± 2.02	0.615	0.000*	0.000*
24 h	11.6 ± 1.85	11.77 ± 2.33	0.346	0.000*	0.000*
FVC(L):					
Baseline	3.89 ± 0.34	3.88 ± 0.31	0.918		
6 h	3.73 ± 0.31	3.63 ± 0.38	0.298	0.000*	0.000*
12 h	3.85 ± 0.33	3.78 ± 0.29	0.368	0.323	0.000*
24 h	3.82 ± 0.33	3.77 ± 0.29	0.500	0.105	0.000*
FEV1(L):					
Baseline	3.96 ± 0.17	3.97 ± 0.18	0.789		
6 h	3.78 ± 0.16	3.74 ± 0.16	0.366	0.000*	0.000*
12 h	3.89 ± 0.16	3.88 ± 0.31	0.881	0.000*	0.059
24 h	3.87 ± 0.16	3.88 ± 0.31	0.909	0.000*	0.069
FEV1/FVC ratio:					
Baseline	1.03 ± 0.11	1.03 ± 0.10	0.881		
6 h	1.02 ± 0.10	1.04 ± 0.12	0.486	0.642	0.390
12 h	1.02 ± 0.10	1.03 ± 0.13	0.581	0.426	0.772
24 h	1.02 ± 0.10	1.04 ± 0.13	0.587	0.624	0.596
PEFR(L/s):					
Baseline	565.27 ± 79.47	531 ± 84.57	0.111		
6 h	555.13 ± 79.05	524.1 ± 85.03	0.149	0.000*	0.000*
12 h	562.87 ± 79.47	524.47 ± 80.85	0.067	0.000*	0.058
24 h	561.53 ± 78.76	525.00 ± 80.77	0.081	0.000*	0.077

Data are presented as mean ± SD *Abbreviations*: standard deviation (*SD*), Diaphragmatic inspiratory Amplitude (*DIA*), forced vital capacity (*FVC*), Forced expiratory volume (*FEV1*), and Peak expiratory flow rate (*PEFR*)., *: Significantly different as *P* < 0.05 P-value1: Comparison between Groups. P-value2: Comparison with Baseline in control Group, P-value3: Comparison with Baseline in RBS Group

Respiratory function tests: FVC declined from preoperative baseline at all postoperative time points in both groups, reaching statistical significance at 6 h in the control group and at 6, 12, and 24 h in the RSB group. In contrast, FEV1 and PEFR showed significant reductions at all time points in the control group, whereas in the RSB group, significant changes were observed only at 6 h. The FEV1/FVC ratio exhibited minimal, nonsignificant changes in both groups. No significant intergroup differences were found for any respiratory parameter at any time point (Table 2).

**Fig. 2:**
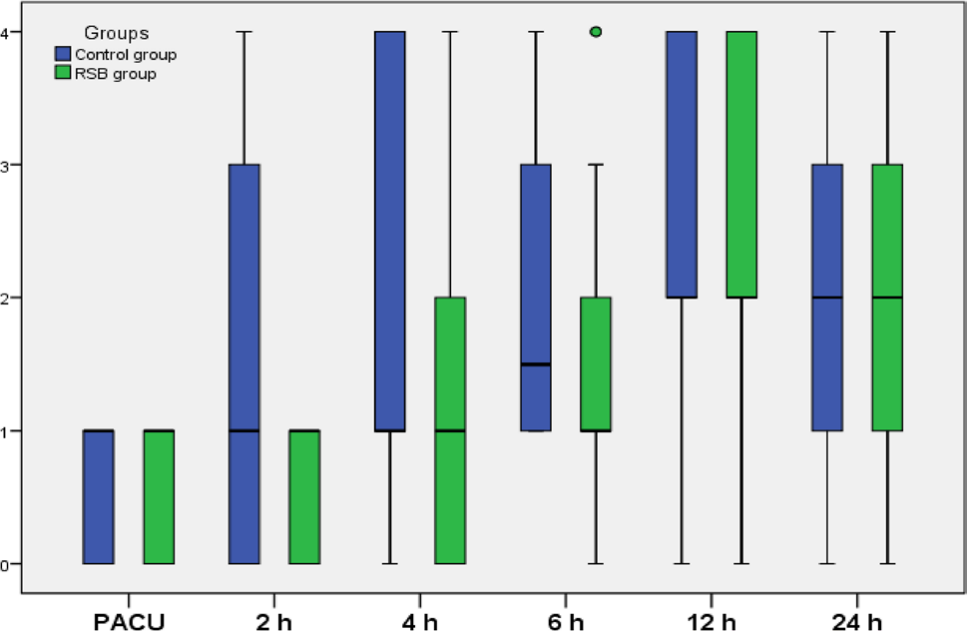
VRS of the studied groups

At 2 h postoperatively, the median VRS score was significantly lower in the RSB group compared with the control group (*P* = 0.007). Thereafter, pain scores increased significantly from baseline in both groups, with no significant differences between groups at subsequent time points (Table 3; Fig. 2).

**Table 3:** Postoperative VRS

	Control group (*n* = 30)	RSB group (*n* = 30)	*P*-value1	*P*-value2	*P*-value3
PACU	1.0 (0.0–1.0)	1.0 (0.0–1.0)	0.797		
2 h	1.0 (0.0–4.0)	1.0 (0.0–1.0)	0.007*	0.002*	1.000
4 h	1.0 (0.0–4.0)	1.0 (0.0–4.0)	0.063	0.001*	0.040*
6 h	1.5 (1.0–4.0)	1.0 (0.0–4.0)	0.074	0.000*	0.001*
12 h	2.0 (0.0–4.0)	2.0 (0.0–4.0)	1.000	0.000*	0.000*
24 h	2.0 (0.0–4.0)	2.0 (0.0–4.0)	1.0001.000	0.000*	0.000*

Data are presented as Median and Range. verbal rating scale (VNRS), hour (hr.). *: Significantly different as P value ≤ 0.05. P-value^1^: Comparison between Groups. P-value^2^: Comparison with PACU in control Group, P-value^3^: Comparison with PACU in RSB Group

Intraoperative fentanyl consumption did not differ significantly between groups. However, the time to first request for rescue analgesia was significantly longer in the RSB group, and total nalbuphine consumption was significantly lower compared with the control group (both *P* < 0.001) (Table 1).

**Fig. 3:**
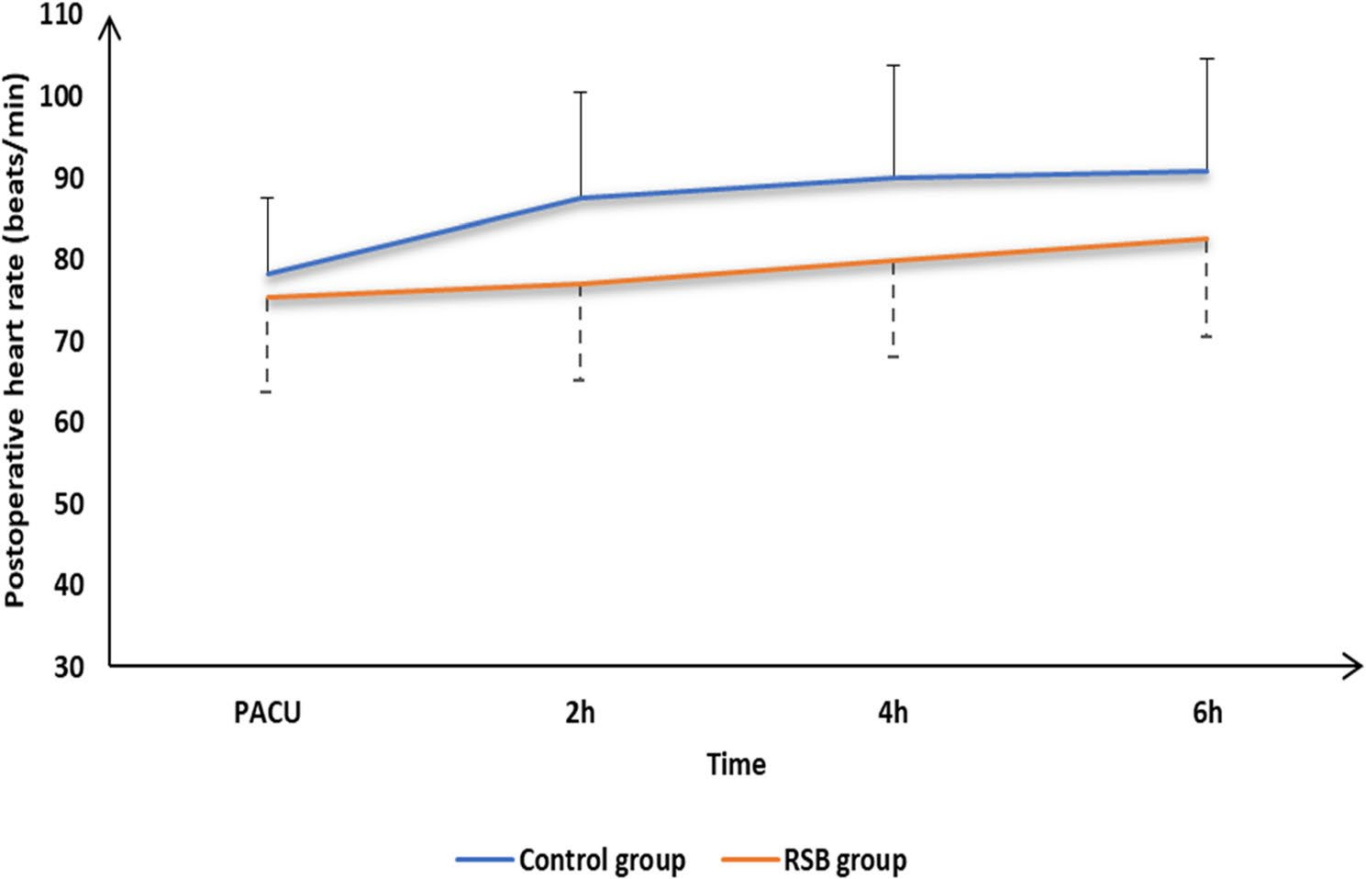
Postoperative heart rate of the studied groups

**Fig. 4:**
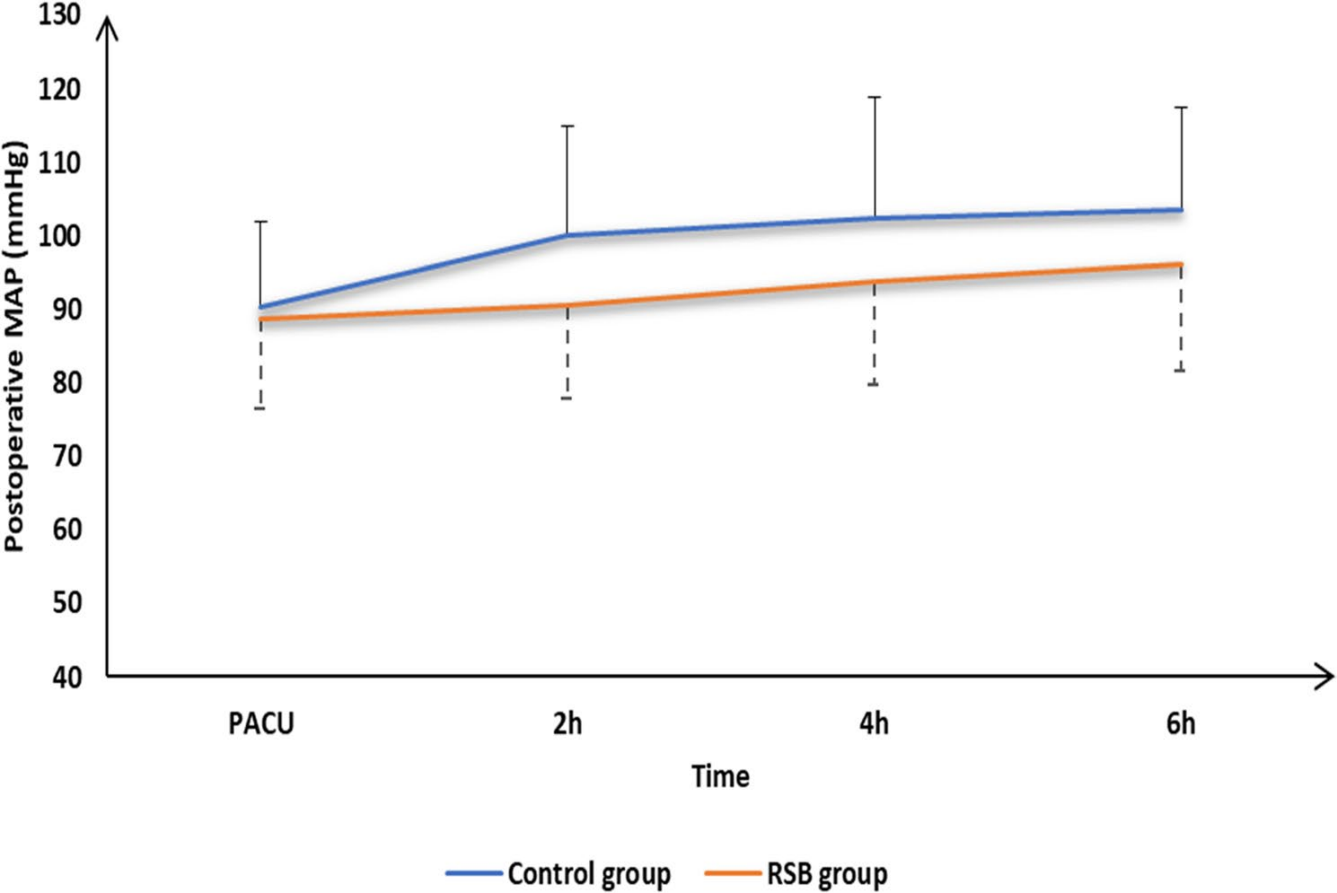
Postoperative mean blood pressure of the studied groups

The incidence of postoperative nausea and vomiting (PONV) was significantly lower in the RSB group (*P* = 0.042). No patients in either group experienced respiratory depression, needle-related trauma, or allergic reactions. Patient satisfaction scores were significantly higher in the RSB group (*P* = 0.041) (Table 1). Hemodynamic parameters remained stable in both groups intraoperatively and throughout the first 24 postoperative hours (Figs. 3 and 4).

## Discussion

In this study, we assessed the respiratory and analgesic benefits of the rectus sheath block (RSB) in patients undergoing upper abdominal surgeries. Our findings demonstrated that RSB provided a clinically acceptable effect in managing postoperative pain. Patients who received RSB exhibited significantly reduced postoperative opioid consumption, which is consistent with previous literature reporting the opioid-sparing effects of regional anesthesia techniques [12, 13]. This reduction in opioid use likely contributed to the stable hemodynamic profile and preservation of respiratory function observed in our study.

The duration of analgesia provided by RSB was found to be sufficient for the immediate postoperative period, enhancing patient comfort without the side effects commonly associated with systemic analgesics. Importantly, despite undergoing upper abdominal surgery, which typically impairs diaphragmatic movement and respiratory parameters [6, 14] all patients maintained diaphragmatic excursion within the normal range postoperatively. Although diaphragmatic excursion decreased in absolute terms compared to preoperative values, this reduction did not differ significantly between the RSB and control groups, suggesting that RSB neither exaggerated nor mitigated the expected diaphragmatic impairment resulting from surgery. The lack of significant differences in diaphragmatic excursion between groups may be influenced by the type and site of surgery, as certain procedures inherently lead to transient diaphragmatic dysfunction regardless of analgesic technique. Additionally, the effect of general anesthesia and intraoperative mechanical ventilation might have contributed to short-term respiratory changes observed in both groups.

Balakrishnan et al., reported a decrease in diaphragmatic inspiratory amplitude following upper abdominal surgery, which was significantly associated with postoperative pulmonary complications [15]. In the same context, Rustagi et al., conclude that DIA significantly declined after pelvic laparoscopic procedures performed in the Trendelenburg position and emphasized that age, duration of anesthesia, and pain following the procedure were significant predictors of the decrease in diaphragmatic excursion [5]. 

In our study, pulmonary function tests, including forced expiratory volume in 1 s (FEV1) and peak expiratory flow rate (PEFR) in RSB group, showed no significant postoperative changes, supporting the observation that respiratory mechanics remained largely intact. Although a reduction in absolute forced vital capacity (FVC) was noted, it was not associated with clinical signs of respiratory depression. This may be attributed to the limited dermatomal spread of RSB, which does not interfere with diaphragmatic innervation, unlike thoracic epidural anesthesia or higher-level blocks. Similarly, Yildiz M, et al. investigated the impact of erector spinae plane (ESP) block on postoperative analgesia and respiratory function following Laparoscopic cholecystectomy, they found improvement in pain scores and significant preservation in (FEV1) and (FVC) in the ESP group in comparison to the control group [16]. Abdel-Ghaffar et al., in their study on patient undergoing elective upper abdominal surgeries who received transversus abdominis plane (TAP) block for postoperative analgesia, reported decreases in postoperative FVC, FEV1, and FVC/FEV1 and the greatest decrease was recorded at 24 h postoperatively [6].

In the present study, although Intraoperative fentanyl consumption was insignificantly different between both groups. Patients who received RSB showed significant lower pain scores with delay in the first request of rescue analgesia after surgery and reduction in the total nalbuphine consumption and correspondingly higher satisfaction than control group.

Several controlled studies [8, 17] on elective laparotomies confirmed a significant decrease in opioid consumption and improved early analgesia with multi-point RSB. Also, a meta-analysis of nine randomized trials in laparoscopic surgery (*N* = 698) demonstrated that RSB significantly reduced 24-hour opioid use and lowered both early rest and movement pain scores without major adverse events [12] These findings reinforce our observation of reduced opioid use and acceptable immediate postoperative pain control., Gurnaney et al., reported RSB combined with TAP provided superior analgesia compared to local infiltration in upper abdominal or major procedures [18].

When compared to thoracic epidurals, Eltwab et al., [19] conclude that RSB offered non-inferior pain control post–cancer surgery, with stable PEFR and fewer hemodynamic fluctuations these suggest that while RSB may not outperform other techniques in isolation, it offers a strong balance of analgesia with fewer side effects. The simplicity of the technique and guidance of ultrasound in this study shared to great extent in the absence of significant related adverse effects (hematomas or needle trauma), also, better pain control, avoidance of morphine reflected significant lowering in PONV in RSB group than control group and use of multimodal analgesia with the choice of nalbuphine (agonist/antagonist) might be responsible for the absence of respiratory depression and pruritus in all studied patient.

Taken together, our results highlight the utility of RSB as a safe and effective component of multimodal analgesia in upper abdominal surgeries. Its use may facilitate early mobilization, reduce reliance on systemic opioids, and preserve postoperative pulmonary function—all of which are critical factors in enhancing recovery after surgery (ERAS) protocols.

## Limitations and future directions

A limitation of this study was that we didn’t specialize the type of abdominal surgery, which was an important predictor of postoperative diaphragmatic excursion. The second limitation was the short follow-up period restricted our ability to assess long-term outcomes related to pain and pulmonary function. Another limitation was absence of sham or placebo block. Also, as a single-center trial, the generalizability of our findings may be limited. Future research should explore the addition of different adjuvants to RSB to prolong its analgesic duration with a longer duration of follow-up and compare the efficacy of RSB in different abdominal procedures.

## Conclusion

Our result reinforced that ultrasound-guided RSB provided effective opioid-sparing analgesia without compromising diaphragmatic or respiratory function. Its simplicity, safety, and compatibility with enhanced recovery after surgery (ERAS) protocols highlight its role as a valuable adjunct to multimodal analgesia in upper abdominal surgery.

## Data Availability

The datasets used and analyzed during the current study are available from the corresponding author upon reasonable request.

## Abbreviations

RSB: Rectus sheath block
DIA: Diaphragmatic inspiratory amplitude
FVC: Forced vital capacity
FEV1: Forced expiratory volume in first second
PEFR: Peak expiratory flow rate
ERAS: Enhanced recovery after surgery
VRS: Verbal rating scale
ASA: American Society of Anesthesiologists
BMI: Body mass index
HR: Heart rate
MAP: Mean arterial blood pressure
PONV: Postoperative nausea and vomiting
PACU: Post-anesthetic care unit
ESP: Erector spinae plane
TAP: Transversus abdominis plane
IV: Intravenous
